# The Role of Gonadotropins and Growth Factor in Regulating Ras During Maturation in Cumulus–Oocyte Complexes of Pigs

**DOI:** 10.3390/ani15142100

**Published:** 2025-07-16

**Authors:** Eunju Seok, Minyoung Son, Seunghyung Lee, Hee-Tae Cheong, Sang-Hee Lee

**Affiliations:** 1 College of Animal Life Sciences, Kangwon National University, Chuncheon 24341, Republic of Korea; yjchsuk@kangwon.ac.kr (E.S.); son021103@kangwon.ac.kr (M.S.); s.lee@kangwon.ac.kr (S.L.); 2 College of Veterinary Sciences, Kangwon National University, Chuncheon 24341, Republic of Korea; htcheong@kangwon.ac.kr; 3 School of ICT, University of Tasmania, Hobart, TAS 7005, Australia

**Keywords:** cumulus–oocyte complexes, Ras, Ras GTPase, maturation, gonadotropin

## Abstract

Oocytes and cumulus cells undergo meiotic resumption and proliferation via gonadotropins and growth factors during maturation, and various small G proteins are activated when COCs undergo physiological changes. This study investigated the influence of gonadotropins and growth factors on Ras and its GTPases during porcine COC maturation. Unmatured COCs were treated with FSH, LH, or EGF for 44 h. The mRNA expression levels of the Ras subfamily (*H-Ras*, *K-Ras*, *N-Ras*, and *R-Ras*), its GTPases (*RASA1* and *SOS1*), and proliferation factors (*ERK*, *CCNB1*, and *Cdc2*) were analyzed using RT-PCR. In contrast to other Ras subfamilies, R-Ras expression is upregulated during COC maturation. We evaluated the effects of FSH, LH, and EGF at various concentrations that most effectively regulated the expression of R-Ras and GTPases. The results demonstrated that 0.5 µg/mL FSH, 10 IU/mL human chorionic gonadotropin (hCG), and 10 ng/mL EGF effectively enhanced R-Ras expression and cell proliferation. FSH supplementation during porcine COC maturation significantly upregulated *R-Ras* and *ERK* expression, independent of LH and EGF, and downregulated *Cdc2* expression. These results indicated that FSH regulates R-Ras expression, thereby promoting cell proliferation during COC maturation. These results provide fundamental knowledge for understanding the role of Ras and its family members in the development of follicular environments in pigs.

## Simple Summary

Some studies have indicated that Ras affects follicular differentiation into the corpus luteum post-ovulation and early embryonic development, but studies on Ras in porcine immature oocytes are limited. In this study, we determined the changes in Ras and its GTPases during maturation of porcine cumulus–oocyte complexes (COCs) following treatment with follicle-stimulating hormone (FSH), luteinizing hormone (LH), and epidermal growth factor (EGF). We demonstrated that gonadotropins and growth factors that induce COC maturation promote cumulus cell proliferation and meiotic resumption in oocytes via Ras-mediated signaling pathways.

## 1. Introduction

Oocytes within the follicles exist in an immature state and are stimulated to mature by various gonadotropins and growth factors, ultimately leading to ovulation [1,2]. At this stage, the oocyte does not exist independently but as a cumulus–oocyte complex (COC) [3,4]. This complex facilitates communication between the oocyte and cumulus cells (CCs) by directly affecting gene expression and protein synthesis, leading to the differentiation and expansion of CCs as well as oocyte maturation [5]. Follicle-stimulating hormone (FSH) promotes the development of immature follicles, while luteinizing hormone (LH) resumes the arrested meiosis of oocytes, leading to their maturation into mature oocytes [6,7]. Furthermore, gonadotropins induce meiosis in arrested oocytes as well as stimulate ovarian cells such as the theca, granulosa, and cumulus cells to produce epidermal growth factor (EGF) [8,9]. The complex of EGF and the EGF receptor (EGFR) induces the proliferation of cumulus cells through the activation of the MAPK/ERK signaling pathway [10,11]. This promotes the rapid proliferation of immature COCs during maturation, along with notable morphological changes, as well as indirectly supporting cumulus cell expansion and nuclear maturation of oocytes [12,13]. Generally, the cell doubling time is 18 h in cumulus cells, and rapid mitosis is maintained for 36–40 h before ovulation in pigs [14]. This specific physiological phenomenon occurs only in reproductive somatic cells with gonadotropin receptors, and their cell signaling differs from that of other somatic cells.

Ras (rat sarcoma virus) is a guanosine triphosphate (GTP)-binding protein involved in various cellular signaling pathways such as cell proliferation, survival, molecular transport, and differentiation [15,16]. The small family of Ras proteins includes H-Ras, K-Ras, N-Ras, and R-Ras, all of which are important in intracellular signaling, trafficking, proliferation, and survival [17,18]. Improper expression of these proteins can lead to cellular pathological conditions. In practice, approximately 27% of all cancers are attributed to the inappropriate expression of Ras proteins [19,20]. Ras functions as a cellular molecular switch, transitioning between its active form, Ras-GTP, and its inactive form, Ras-guanosine diphosphate (GDP), and regulates various cellular processes [17,21]. When the inactive form, Ras-GDP, transitions to the active form, Ras-GTP, Ras is facilitated by the exchange of GDP for GTP by a protein called the guanine nucleotide exchange factor (GEF). Conversely, Ras-GTP is hydrolyzed to Ras-GDP with the assistance of Ras-GTPase-activating protein (GAP) [17,22]. Therefore, disruption in the regulation of Ras activity/inactivity by GEF and GAP can result in negative implications for cellular pathology. For example, if Ras remains persistently activated owing to the overexpression of Ras GEF or the loss of function of Ras GAP, cellular carcinogenesis can be induced [23]. Ras is a well-known oncogene, and several studies have focused on tumorigenesis caused by Ras mutations [24,25]. However, studies on Ras expression in oocytes and ovaries are limited. Although some studies have indicated that Ras affects follicular differentiation into corpus luteum post-ovulation [26] and early embryonic development [27], studies on Ras in porcine immature oocytes are limited.

We hypothesized that Ras and its regulators (GEFs and GAPs) would be involved in rapid proliferation during the maturation period of porcine COCs and that the interaction between G-protein coupled receptors and gonadotropins or growth factors involved in COC maturation could potentially activate Ras and Ras GTPase. Therefore, we determined the changes in Ras and its GTPases during porcine COC maturation and investigated the influence of FSH, LH, and EGF on Ras signaling and proliferation-related factors during porcine COC maturation.

## 2. Materials and Methods

### 2.1. Collection of Porcine COCs

The porcine ovarian samples used in the experiment were collected from immature gilt in the slaughterhouse (Pochen farm, Pocheon, Republic of Korea) and transported to the laboratory in 0.9% saline solution at 38.5 °C within 2 h. Follicular fluid measuring 3–6 mm was aspirated using an 18-gauge needle after three washes with 0.85% saline solution. The COCs were washed three times with phosphate-buffered saline with polyvinyl alcohol (PBS-PVA), collected in the follicular fluid under a stereo microscope (SZX16, Olympus Tokyo, Japan), and COCs (50–60 per sample and a total of six replication) were cultured in 650 µL tissue culture medium 199 (TCM-199; Invitrogen, Carlsbad, CA, USA) with 0.1% PVA (Sigma, St. Louis, MO, USA), 0.57 mM cysteine (Sigma), 10 ng/mL EGF (Sigma), 10% porcine follicular fluid (pFF), 0.5 µg/mL FSH (Sigma), and 10 IU/mL human chorionic gonadotropin (hCG; Intervet, The Netherlands) for 22 h at 38.5 °C in 5.0% CO_2_. The collected COCs exhibiting a homogenous ooplasm and 3 to 4 compact layers of cumulus cells were selected for IVM [28]. The culture medium was replaced with a hormone-free TCM-199 culture medium, and further incubation was conducted for an additional 22 h at 38.5 °C in a 5.0% CO_2_ environment [29]. The degree of cumulus expansion in COCs was assessed based on their morphological characteristics at 0, 22, and 44 h after the initiation of in vitro maturation (IVM), and the area of COCs was quantitatively measured using ImageJ 1.54p software (NCBI, Bethesda, MD, USA). The relative mRNA expression levels were normalized in the 0 h treatment group.

### 2.2. Treatment with FSH, LH, and EGF

The COCs were collected and washed three times with PBS-PVA before being transferred to a fresh culture medium consisting of TCM-199 supplemented with 1.0% pFF. The COCs that exhibited a homogenous ooplasm and 3 to 4 compact layers of cumulus cells were selected for IVM [28]. Each experiment was performed in four replicates. To assess the independent roles of FSH, LH, and EGF in COC (30–40 per sample and a total of four replications) maturation, the cultures were treated with varying concentrations of FSH (0.0, 0.1, 0.5, and 1.0 µg/mL), hCG (0.0, 1.0, 10, and 20 IU/mL), and EGF (0.0, 1.0, 10, and 20 ng/mL) for a total incubation period of 44 h. The minimum and maximum effective concentrations of these hormones and growth factors for porcine COC maturation were determined using previously established methods [30,31]. Subsequently, additional COCs were cultured in 500 µL of TCM-199 medium with FSH, hCG, and EGF at 38.5 °C in a 5.0% CO_2_ environment for 44 h.

### 2.3. Quantitative Reverse Transcription PCR

Total RNA was extracted from the COCs using an easy-spin Total RNA Extraction kit (iNtRON Biotechnology, Inc., Seongnam-si, Republic of Korea). The RNA concentration was measured using an EzDrop 1000C spectrophotometer (Blue-Ray Biotech, New Taipei City, Taiwan). Based on the measured RNA concentration, cDNA was synthesized using the PrimeScript™ 1st strand cDNA Synthesis Kit (Takara, Tokyo, Japan). PCR was performed using Veriti 96-well Thermal Cycler (Applied Biosystems, Waltham, MA, USA) with AccuPower^®^ PCR PreMix & Master Mix (Bioneer, Seoul, Republic of Korea), following the manufacturer’s protocol. Gene expressions of gonadotropin and growth factor receptors (*FSHR*, *LHR*, and *EGFR*), Ras subfamily (*H-Ras*, *K-Ras*, *N-Ras*, and *R-Ras*), Ras GEFs (*RasGEF-1B* and *SOS1*), Ras GAPs (*RasGAP* and *RASA1*), and proliferation factors (*ERK*, *CCNB1*, and *Cdc2*) were analyzed according to the PCR conditions (Table 1). The amplified products were separated by electrophoresis on a 1.5% agarose gel containing RedSafe Nucleic Acid Staining Solution (iNtRON) at 100 V for 20 min and photographed under UV illumination. Relative mRNA expression levels were normalized to β-actin, and ImageJ software (NCBI) was used for image analysis. The primers are listed in Table 1.

### 2.4. Statistical Analysis

Each experiment was repeated at least thrice. Data are presented as mean ± standard error. Data were analyzed using *t*-test or one-way analysis of variance (ANOVA) followed by Tukey’s multiple comparison test, using SAS ver. 9.4 software. Differences were considered statistically significant at *p* < 0.05.

## 3. Results

### 3.1. Changes in Ras and Its Regulators During Maturation in Porcine COCs

**Figure 1:**
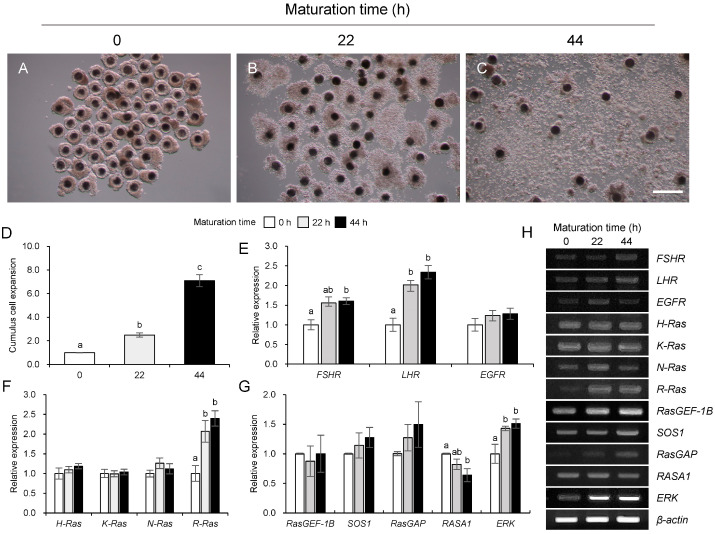
Changes in Ras subfamily and it GTPase genes during maturation in porcine cumulus–oocyte complexes (COCs). Morphology of COCs at 0 h (**A**), 22 h (**B**), and 44 h (**C**) of maturation. The relative expansion of cumulus cell size during the maturation in porcine COCs (**D**). Expression of gonadotropin receptors (*FSHR* and *LHR*) and epidermal growth factor (*EGFR*) genes (**E**), Ras subfamily (*H-Ras*, *K-Ras*, *N-Ras*, and *R-Ras*) genes (**F**), Ras GEF (*RasGEF-1B* and *SOS1*), Ras GAP (*RasGAP* and *RASA1*), and *ERK* genes (**G**) during maturation. Representative RT-PCR gel image showing the mRNA expression of all analyzed genes (**H**). a–c Bars with different letters are significantly different (*p* < 0.05, *n* = 6). Scale bar = 500 μm.

At 0 h of maturation, cumulus cells formed a compact structure around the oocyte (Figure 1A). Subsequently, at 22 h of maturation, a notable increase in cumulus cell proliferation was observed (Figure 1B). Cumulus cells exhibited significant proliferation and expansion (Figure 1C) at 44 h of maturation. The expansion of cumulus cells significantly (*p* < 0.05) increased during the maturation of porcine COCs (Figure 1D). *FSHR* mRNA expression was significantly (*p* < 0.05) higher at 44 h than at 0 h (Figure 1E) of maturation in the COCs. The expression of *LHR* mRNA significantly (*p* < 0.05) increased at 22 and 44 h compared to that at 0 h of maturation; however, no significant difference was observed between 22 and 44 h (Figure 1E). Conversely, *EGFR* mRNA expression levels did not differ significantly throughout COC maturation (Figure 1E). Changes in the expression levels of the Ras family genes were measured according to the maturation time of COCs, as shown in Figure 1F,H. For *R-Ras* mRNA, a significant (*p* < 0.05) increase was observed in its expression as the maturation time (22 and 44 h) of porcine COCs progressed (Figure 1F). Conversely, no significant differences were observed in expression levels according to the maturation time of porcine COCs for *H-Ras*, *K-Ras*, and *N-Ras* mRNAs (Figure 1F). Additionally, the mRNA expression levels of *RasGEF-1B* and *SOS1* (Ras GEF), *RasGAP* and *RASA1* (Ras GAP), and *ERK* were measured during the maturation of porcine COCs (Figure 1G). During the maturation of porcine COCs, the mRNA expression of *SOS1* remained unchanged, whereas that of *RASA1* significantly decreased (*p* < 0.05) as maturation advanced (Figure 1G). *ERK* expression of *ERK* significantly increased (*p* < 0.05) as COC maturation progressed (Figure 1G).

### 3.2. Influences of FSH on the Expression of Ras and Its Regulator Genes in Porcine COCs

**Figure 2:**
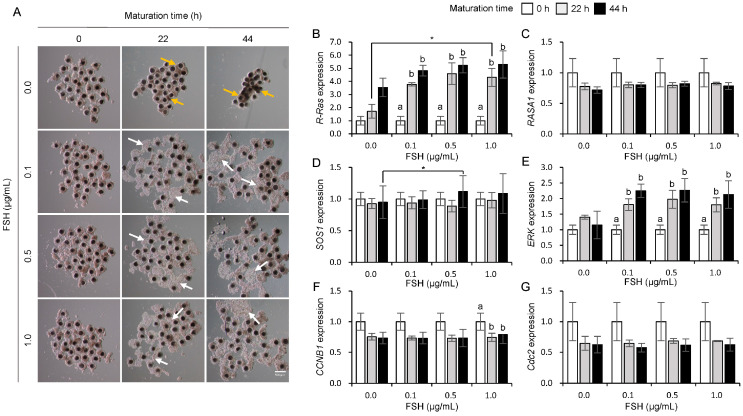
Influence of follicle stimulating hormone (FSH) on morphological changes (**A**) and mRNA expression of *R-Ras* (**B**), *RASA1* (**C**), *SOS1* (**D**), *ERK* (**E**), *CCNB1* (**F**), and *Cdc2* (**G**) during maturation in porcine cumulus–oocyte complexes (COCs). White arrows indicate expanded cumulus cells, and yellow arrows indicate shrunken cumulus cells. a, b Bars with different letters indicate significant differences in gene expression levels within the same concentration. An asterisk (*) indicates significant differences in gene expression levels between different concentration treatment groups (*p* < 0.05, *n* = 4). Scale bar = 500 μm.

Figure 2A shows the morphological changes in COCs during maturation in response to FSH treatment. The size of cumulus cells increased in the 0.1, 0.5, and 1.0 µg/mL FSH treatment groups (Figure 2A, white arrows); however, the control group (0.0 µg/mL FSH, Figure 2A, yellow arrows) did not increase at 22 and 44 h of maturation in porcine COCs. At both 22 h and 44 h, R-Ras mRNA expression showed a concentration-dependent increase, with the 1.0 µg/mL FSH treatment resulting in a significant increase (*p* < 0.05) in *R-Ras* mRNA expression at 22 h of maturation in COCs (Figure 2B). However, *RASA1* mRNA expression levels did not differ with the FSH concentration (Figure 2C). The *SOS1* expression at 44 h of COC maturation significantly increased (*p* < 0.05) in the 0.5 µg/mL FSH treatment group compared to that in the 0.0 µg/mL FSH treatment group (Figure 2D). The *ERK* mRNA expression significantly increased (*p* < 0.05) at 22 and 44 h compared to that at 0 h when COCs were treated with 0.1, 0.5, and 1.0 µg/mL FSH (Figure 2E). The expression of *CCNB1* mRNA significantly decreased (*p* < 0.05) at 22 and 44 h compared to that at 0 h in the 1.0 µg/mL FSH treatment group (Figure 2F). In contrast, no significant differences was observed in *Cdc2* expression across the FSH treatment group (Figure 2G).

### 3.3. Influences of hCG on the Expression of Ras and Its Regulator Genes in Porcine COCs

**Figure 3:**
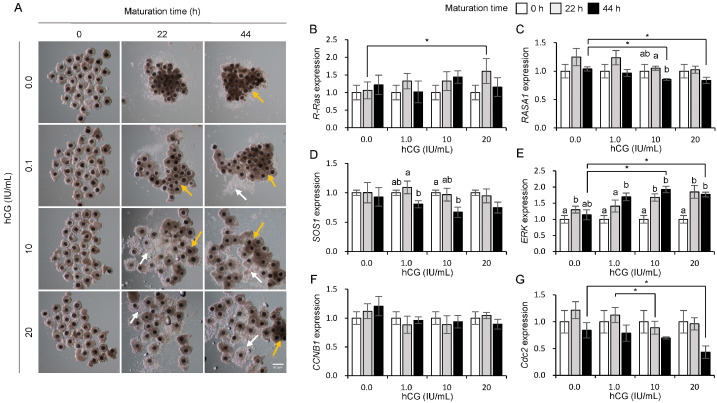
Influence of human chorionic gonadotropin (hCG) on morphological changes (**A**) and mRNA expression of *R-Ras* (**B**), *RASA1* (**C**), *SOS1* (**D**), *ERK* (**E**), *CCNB1* (**F**), and *Cdc2* (**G**) during maturation in porcine cumulus–oocyte complexes (COCs). White arrows indicate expanded cumulus cells, and yellow arrows indicate shrunken cumulus cells. a, b Bars with different letters indicate significant differences in gene expression levels within the same concentration. An asterisk (*) indicates significant differences in gene expression levels between different concentration treatment groups (*p* < 0.05, *n* = 4). Scale bar = 500 μm.

Figure 3A shows the morphological changes observed in the COCs during maturation in response to hCG treatment. After 22 h of hCG treatment, most COCs in the 0 and 0.1 IU/mL groups exhibited a shrunken morphology (Figure 3A, yellow arrows). In contrast, the 10 and 20 IU/mL hCG-treated groups showed shrunken (Figure 3A, yellow arrows) and expanded (Figure 3A, white arrows) COCs. At 44 h of maturation, most COCs in the 0 IU/mL hCG group exhibited shrunken morphology (Figure 3A, yellow arrows). However, the 0.1, 10, and 20 IU/mL hCG groups showed both shrunken (Figure 3A, yellow arrows) and expanded (Figure 3A, white arrows) forms, with a greater proportion of expansion observed particularly in the 10 and 20 IU/mL groups. As shown in Figure 3B, no significant changes in *R-Ras* expression were observed across different hCG concentrations during COC maturation. However, *RASA1* (Figure 3C) and *SOS1* (Figure 3D) expression significantly (*p* < 0.05) decreased at 44 h of oocyte maturation in the 10 IU/mL hCG-treated group. Notably, at 44 h of maturation, *RASA1* expression was significantly reduced in the 10 and 20 IU/mL hCG treatment groups compared to the 0.0 IU/mL control group (*p* < 0.05). A clear concentration-dependent increase in *ERK* mRNA expression was observed (Figure 3E), with the 10 and 20 IU/mL hCG groups showing significantly higher levels at 44 h than the 0.0 IU/mL group (*p* < 0.05). In contrast, *CCNB1* (Figure 3F) and *Cdc2* (Figure 3G) did not show significant changes in expression at different maturation stages after treatment with hCG alone.

### 3.4. Influences of EGF on the Expression of Ras and Its Regulator Genes in Porcine COCs

**Figure 4:**
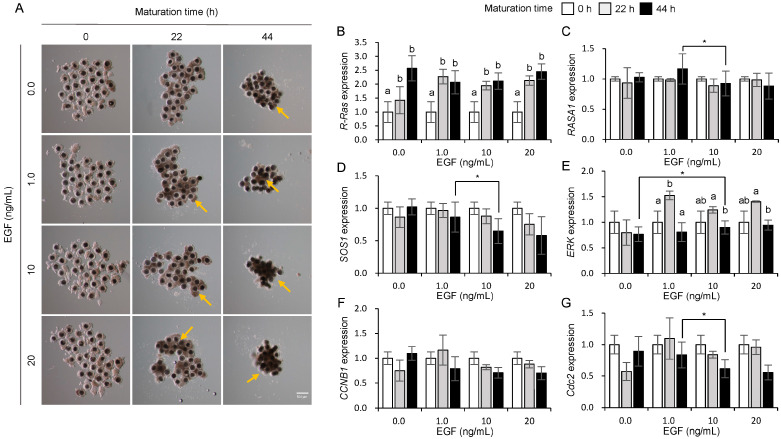
Influence of epidermal growth factor (EGF) on morphological changes (**A**) and mRNA expression of *R-Ras* (**B**), *RASA1* (**C**), *SOS1* (**D**), *ERK* (**E**), *CCNB1* (**F**), and *Cdc2* (**G**) during maturation in porcine cumulus–oocyte complexes (COCs). Yellow arrows indicate shrunken cumulus cells. a, b Bars with different letters indicate significant differences in gene expression levels within the same concentration. An asterisk (*) indicates significant differences in gene expression levels between different concentration treatment groups (*p* < 0.05, *n* = 4). Scale bar = 500 μm.

Figure 4A shows the morphological changes observed in the COCs during maturation in response to EGF treatment. A more compact and aggregated morphology (Figure 4A, yellow arrows) of COCs was observed in the 1.0, 10, and 20 ng/mL EGF treatment groups, whereas such morphological changes were not apparent in the control group (0.0 ng/mL EGF, Figure 4A) at 22 and 44 h of maturation in porcine COCs. As shown in Figure 4B, *R-Ras* expression significantly increased (*p* < 0.05) in the 1.0, 10, and 20 ng/mL EGF treatment groups at 22 and 44 h compared with that at 0 h (Figure 4B). However, Figure 4C,D indicate that the expression of *RASA1* and *SOS1* at 44 h significantly decreased (*p* < 0.05) in the group treated with 10 ng/mL EGF compared to the other groups. *ERK* (Figure 4E) expression significantly increased (*p* < 0.05) during the early phase (0–22 h) but decreased during the late phase (22–44 h) of oocyte maturation in the 1.0, 10, and 20 ng/mL EGF treatment groups, possibly as a regulatory mechanism to control excessive cell proliferation induced by R-Ras upregulation. In particular, a significant increase in ERK expression was observed at 44 h in the 10 ng/mL EGF-treated group compared to the 0.0 ng/mL group (*p* < 0.05). The expression of *CCNB1* mRNA showed no significant difference (Figure 4F), whereas *Cdc2* expression significantly decreased at 44 h in the 10 ng/mL EGF treatment group (Figure 4G).

### 3.5. Mechanism of Ras Regulation by Gonadotropins and EGF

**Figure 5:**
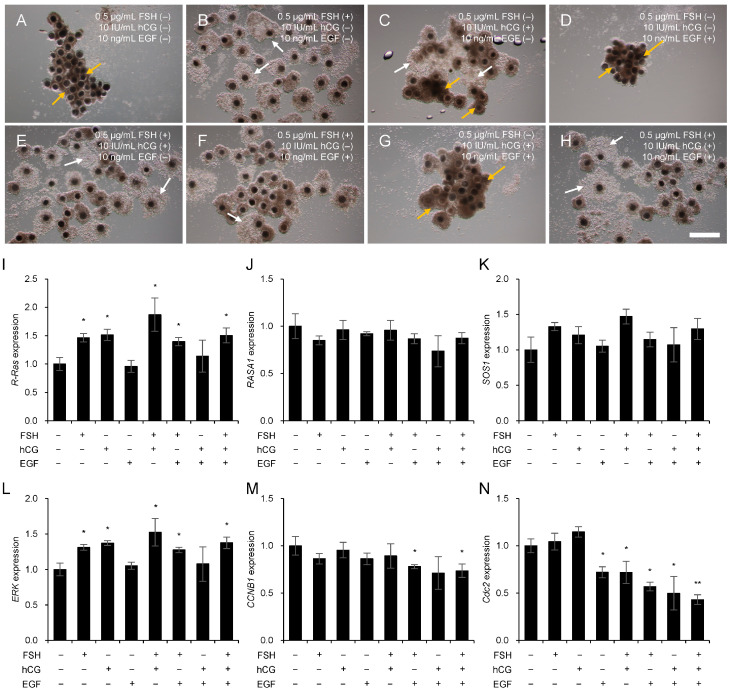
Effects of follicle stimulating hormone (FSH), human chorionic gonadotropin (hCG), and epidermal growth factor (EGF) on morphological changes (**A**–**H**) and mRNA expression of *R-Ras* (**I**), *RASA1* (**J**), *SOS1* (**K**), *ERK* (**L**), *CCNB1* (**M**), and *Cdc2* (**N**) during maturation in porcine cumulus–oocyte complexes (COCs). The treatment concentrations of FSH, hCG, and EGF were determined based on the results of previous experiments, and the COCs were treated with a combination of 0.5 μg/mL FSH, 10 IU/mL hCG, and 10 ng/mL EGF. White arrows indicate expanded cumulus cells, and yellow arrows indicate shrunken cumulus cells. An asterisk (*) indicates significant differences in gene expression levels between different concentration treatment groups (*p* < 0.05, *n* = 4). Scale bar = 500 μm.

Based on the previous results, 0.5 µg/mL FSH, 10 IU/mL hCG, and 10 ng/mL EGF were used to determine changes in Ras and its regulator genes in porcine COCs. Figure 5 shows the expression changes in Ras, Ras GTPase regulators, and cell proliferation factors during porcine COC maturation, depending on the presence or absence of FSH, hCG, and EGF. Figure 5A shows the morphological changes in porcine COCs during maturation with or without gonadotropin and EGF treatment. Cumulus cell expansion was observed in treatment groups supplemented with FSH and hCG (Figure 5A, white arrows). In contrast, shrinkage of cumulus cells was observed in the EGF treatment group without FSH supplementation (Figure 5A, yellow arrows). *R-Ras* mRNA expression was significantly increased (*p* < 0.05) in the gonadotropin-treated groups, particularly in the presence of FSH (Figure 5B). In contrast, no significant changes were observed in *RASA1* (Figure 5C) or *SOS1* (Figure 5D) levels, regardless of gonadotropin or growth factor treatment. Similarly to R-Ras, *ERK* mRNA expression was significantly upregulated (*p* < 0.05) in the gonadotropin-treated groups, particularly in the presence of FSH (Figure 5E). The mRNA expression levels of *CCNB1* (Figure 5F) and *Cdc2* (Figure 5G) significantly decreased (*p* < 0.05) after the combined treatment with gonadotropins and growth factors.

## 4. Discussion

The Ras subfamily is the founding member of a large superfamily of small monomeric GTPases that regulate diverse cellular processes [32]. The transition between the active GTP-bound and inactive GDP-bound states of small GTPases is controlled by GEFs, which stimulate the exchange of GDP with GTP, and by GAPs, which terminate the active state by stimulating GTP hydrolysis [23,33]. The Ras/MAPK/*ERK* signaling pathway is crucial in cell proliferation, differentiation, and oocyte maturation, mainly through the activation of receptor tyrosine kinases (RTKs), such as EGFR [34,35]. In the ovarian follicles, FSH and LH are essential for the proliferation, differentiation, and maturation of COCs [36,37,38]. FSH is particularly critical for stimulating the expression of EGF-like factors, which then activate *ERK*1/2 signaling in cumulus cells, leading to cumulus expansion and oocyte meiotic resumption [39,40]. During ovulation, LH binds to its receptor (LHR) and induces the release of EGFR ligands, further activating the *ERK*/MAPK pathway and promoting the resumption of meiosis in oocytes [5,41]. The binding of EGF to EGFR and RTK subsequently initiates multiple downstream signaling pathways, including MAPK and PI3K, which mediate cumulus cell expansion and meiotic progression, as well as follicular development and ovulation [12,42,43,44]. Although these pathways have been studied extensively, the role of Ras in porcine COC maturation remains unclear.

In this study, we investigated changes in the expression of Ras and Ras GTPases during porcine COC maturation. Our analysis of *H-Ras*, *K-Ras*, *N-Ras*, and *R-Ras* expression levels revealed that only *R-Ras* exhibited significant changes during maturation. Additionally, *ERK* expression was upregulated in response to Ras activation, whereas *RasGAP* expression progressively decreased as the COCs matured. These findings indicate that R-Ras functions as a specific regulatory factor during porcine COC maturation. Although *K-Ras* expression has been previously identified in mouse ovaries [45], no studies have investigated the changes in R-Ras expression during porcine COC maturation.

To further investigate the role of R-Ras in COC maturation, we examined the interaction between Ras signaling and gonadotropins or growth factors within the follicular environment. To identify the optimal concentrations for the effective modulation of Ras and Ras GTPase expression, COCs were cultured with various concentrations of FSH. The results demonstrated that *R-Ras* and *ERK* expression significantly increased in all FSH-treated groups. These results support previous findings that FSHR inhibition reduces Ras production in sheep COCs [46]. Although *SOS1* expression did not exhibit notable changes across FSH-treated groups, a significant upregulation was observed in the group treat with 0.5 µg/mL FSH compared to the untreated control. These results indicate that the FSH-induced Ras upregulation likely contributed to increased *ERK* expression, but this alone may not be sufficient for complete oocyte maturation and development. Subsequently, COCs were treated with a range of hCG concentrations. Although *R-Ras* expression remained unchanged across different hCG concentrations, the group treated with 10 IU/mL hCG exhibited significant alterations in *SOS1* and *RASA1* expression. Notably, despite the absence of changes in *R-Ras* expression, hCG treatment resulted in increased *ERK* expression, which we hypothesized was mediated by observed variations in *RASA1* and *SOS1* expression. This finding is consistent with previous studies that reported that the absence of *FSHR* in immature granulosa cells reduces *LHR* mRNA transcription levels, thereby impairing follicular maturation, and that the LH surge can transiently activate the Ras-ERK signaling pathway [47]. Based on these results, 10 IU/mL of hCG was the most effective concentration for modifying Ras GTPase expression. Additionally, treatment with EGF at various concentrations revealed that the most pronounced differences in *R-Ras* and *ERK* expression were observed in the 10 ng/mL EGF-treated group. This result is consistent with the established role of EGF as a general regulator of the Ras/ERK signaling pathway [48,49,50]. However, the expressions of *RASA1* and *SOS1* decreased in the 10 ng/mL EGF-treated group. This result indicates that the decrease in GEF expression is due to the negative feedback induced by the upregulation of R-Ras, and the reduction in GAP is likely attributable to signaling deficiencies caused by excessive R-Ras expression.

During porcine COC maturation, *R-Ras* gene expression was modulated in the presence or absence of gonadotropin. Although we did not confirm differences in Ras and Ras GTPase expression at the protein level, this study suggests that Ras signaling may be important in mediating gonadotropin- and growth factor-induced oocyte maturation and cumulus cell expansion. Considering that Ras activity is primarily regulated at the posttranslational level, further validation through protein-level analyses is essential. In future studies, we plan to establish oocyte models with regulated Ras signaling and subsequently investigate the effects of hormone treatments to further elucidate the signal transduction mechanisms involved in oocyte development, as well as to clarify the role of R-Ras in porcine follicular development.

Based on these findings, we analyzed the expression changes of Ras and Ras GTPases during porcine COC maturation following individual or combined treatments with FSH (0.5 µg/mL), hCG (10 IU/mL), and EGF (10 ng/mL). Notably, regardless of the addition of hCG or EGF, all FSH-treated groups exhibited increased *R-Ras* expression and significant upregulation of *ERK* expression. These findings strongly indicate that gonadotropin-mediated Ras signaling is intricately involved in follicular maturation. The expression levels of *SOS1* and *RASA1* were not significantly altered, possibly because these factors regulate Ras activity through protein–protein interactions [51] rather than de novo synthesis or degradation, making precise gene-level analysis challenging. Additionally, the cell cycle regulators *CCNB1* and *Cdc2* exhibited changes in expression in response to FSH, hCG, and EGF treatments, suggesting that Ras signaling during COC maturation may influence cumulus cell proliferation and the resumption of meiosis.

This study effectively captured the specific expression dynamics of R-Ras during porcine COC maturation and elucidated a molecular regulatory mechanism by which R-Ras is modulated by gonadotropins and growth factors within the follicular environment. Although these results highlight the potential role of R-Ras, further investigation is necessary to evaluate its functional activity at the protein level, considering that Ras and Ras GTPases regulate their activity through conformational changes between GTP- and GDP-bound states [52]. Despite these limitations, our study provides compelling genetic evidence that FSH directly affects Ras expression. The findings of this study contribute to reproductive physiology and medicine by elucidating the regulatory pathway for Ras signaling. This study advances our understanding of hormonal regulation in the reproductive system and contributes to the development of therapeutic approaches for infertility and ovarian dysfunction.

## 5. Conclusions

In conclusion, this study effectively captured *R-Ras*-specific signaling events during porcine COC maturation. We demonstrated that gonadotropins and growth factors that induce COC maturation promote cumulus cell proliferation and meiotic resumption in oocytes via Ras-mediated signaling pathways. These findings provide novel insights into the molecular control of follicular maturation and indicate that *R-Ras* is a key regulator.

## Figures and Tables

**Table 1:** Information on primer condition.

Genes	Sequence (5′–3′)	Annealing Temp. (°C)	Cycle	Product Size (bp)
*β-actin*	F: GGACTTCGAGCAGGAGATGG	60	30	233
	R: GCACCGTGTTGGCGTAGAGG			
*FSHR*	F: GATCCTGATCACCAGCCAATAC	60	32	217
	R: GACTGAGAGCTCACTAGCAAAG			
*LHR*	F: CTGTCCTCTTTGTTCTCCTGAC	60	32	191
	R: AGCAACACTACACCCATTCC			
*EGFR*	F: CCTTGGGAACTTGGAGATCACCTAC	60	32	344
	R: TGTTGCTTAGAAAGTCGCTGTTGAC			
*H-Ras*	F: CGCCATCAACAACACCAAATC	60	32	194
	R: TGGCTGAGGTCTCGATGTAA			
*K-Ras*	F: CAATGAGGGACCAGTACATGAG	60	32	205
	R: GCTAAGTCCTGAGCCTGTTT			
*N-Ras*	F: TACAAACTGGTGGTGGTTGG	60	32	219
	R: GCTAAGTCCTGAGCCTGTTT			
*R-Ras*	F: CCCACTATTGAAGACTCCTACAC	60	32	205
	R: GAAGTCATCTCGGTCCTTGAC			
*RasGEF-1B*	F: GGCAGCACAAAGGTCTTTAAC	60	35	201
	R: GGACACTCCACTTGTTTCCA			
*SOS1*	F: CTGCTCACCTTACACCCAATAG	60	35	231
	R: CACCACAGCTACCCTTTCTT			
*RasGAP*	F: CAAACGCACGAAGTCACAAC	60	35	203
	R: CTGGCTTGATGATGGAGTCTT			
*RASA1*	F: TACTTCCACCGACACTGAGATA	60	35	221
	R: CTGCACAGACTTAGCCACTAAT			
*ERK*	F: GACCAGCTCAACCACATTCT	60	32	213
	R: CCGACAGAAGCCAAGATAACA			
*CCNB1*	F: GTGTCAGGCTTTCTCTGATGT	60	32	199
	R: CCAGTCAATTAGGATGGCTCTC			
*Cdc2*	F: GGTGTTCCTAGTACTGCCATTC	60	32	179
	R: GAATCCATGAACTGACCAGGAG			

## Data Availability

All data generated or analyzed during this study are included in this published article.
